# Supplementary material for: Self-amplifying COVID-19 mRNA vaccination induces longitudinally enhanced antibody function in a Phase 3 trial
Source: NPJ Vaccines. 2026 Mar 30;11:106. doi: 10.1038/s41541-026-01431-x (PMC13199437; doi:10.1038/s41541-026-01431-x)
Supplement: Supplementary file 1 — Supplementary informationn. [file 41541_2026_1431_MOESM1_ESM.pdf]

Supplemental Information

Supplementary information includes Supplementary Table 1 and Supplementary Figures 1-9 and their corresponding captions.

Supplementary Table 1

REAGENT or RESOURCE	SOURCE	IDENTIFIER
**Antibodies**		
Mouse Anti-Human IgG Fc-PE (JDC-10)	SouthernBiotech	9040-09
Mouse Anti-Human IgG1 Hinge-PE (4E3)	SouthernBiotech	9052-09
Mouse Anti-Human IgG2 Fc-PE (31-7-4)	SouthernBiotech	9060-09
Mouse Anti-Human IgG3 Hinge-PE (HP6050)	SouthernBiotech	9210-09
Mouse Anti-Human IgG4 Fc-PE (HP6025)	SouthernBiotech	9200-09
Mouse Anti-Human IgA1-PE (B3506B4)	SouthernBiotech	9130-09
Mouse Anti-Human IgA2-PE (A9604D2)	SouthernBiotech	9140-09
Mouse Anti-Human IgM-PE (SA-DA4)	SouthernBiotech	9020-09
PE-Cy™5 Mouse Anti-Human CD107a	BD Biosciences	555802
PE-Cy™7 Mouse Anti-Human CD56 (NCAM-1)	BD Biosciences	557747
APC-Cy™7 Mouse Anti-Human CD16	BD Biosciences	557758
Pacific Blue™ Mouse Anti-Human CD3	BD Biosciences	558117
PE Mouse Anti-Human MIP-1β	BD Biosciences	550078
FastImmune™ FITC Mouse Anti-Human IFN-γ	BD Biosciences	340449
Pacific Blue™ anti-human CD14 Antibody	BioLegend	325616
FITC IgG Goat Anti-Guinea Pig Complement C3	MP Biomedicals	0855385
**Chemicals, Peptides, and Recombinant Proteins**		
SARS-CoV-2 WT Spike Trimer	Sino Biological	40589-V08H4
SARS-CoV-2 WT Spike Trimer	Abwiz Bio	2720-200
SARS-CoV-2 KP.3 Spike Trimer	Abwiz Bio	2804-200
SARS-CoV-2 Delta Spike Trimer	Sino Biological	4089-V08B16
SARS-CoV-2 Delta Spike Trimer	Abwiz Bio	2611-200
SARS-CoV-2 BA.2 Spike Trimer	Abwiz Bio	2460-200
SARS-CoV-2 BA.5 Spike Trimer	Abwiz Bio	2688-200
SARS-CoV-2 XBB.1.5 Spike Trimer	Abwiz Bio	2712-200
Influenza A H1NA (A/Brisbane/59/2007) Hemagglutinin	Sino Biological	11052-V08H
Human Cytomegalovirus Glycoprotein B	Sino Biological	10202-V08H1
Ebola Virus (subtype Zaire) Glycoprotein	Sino Biological	40459-V08H

Human soluble FcγRIIA	Duke University	Custom Order
Human soluble FcγRIIB	Duke University	Custom Order
Human soluble FcyRIIIA	Duke University	Custom Order
Human soluble FcyRIIIB	Duke University	Custom Order
Sulfo-NHS (N-hydroxysulfosuccinimide)	Thermo Fisher	A39269
Pierce EDC	Thermo Fisher	A35391
BirA500: BirA biotin-protein ligase standard reaction kit	Avidity LLC	BirA500
Streptavidin-R-Phycoerythrin	Agilent	PJ31S
Brefeldin A	Sigma Aldrich	B7651
Human IL-15 Recombinant Protein	Stemcell Technologies	78031
Gelatin Veronal Buffer	Sigma Aldrich	G6514
Paraformaldehyde solution 4% in PBS	Santa Cruz Biotechnology	sc-281692
GolgiStop™ Protein Transport Inhibitor	BD Biosciences	554724
UltraPure™ 0.5M EDTA, pH 8.0	Thermo Fisher	15575020
Dimethyl Sulfoxide, Fisher BioReagents™	Fisher Scientific	BP231
RPMI-1640 Medium	Sigma Aldrich	R0883
HEPES Buffer	Fisher Scientific	MT25060CI
Trypan Blue Solution, 0.4% (w/v) in PBS	Fisher Scientific	MT25900CI
L-glutamine Solution	Fisher Scientific	MT25005CI
Penicillin-Streptomycin Solution	Fisher Scientific	MT30001CI
Fetal Bovine Serum	Sigma Aldrich	F4135
Bovine Serum Albumin	Sigma Aldrich	A4737
Polysorbate 20	Fisher Scientific	BP337
Dulbecco's Phosphate-Buffered Salt Solution 1X	Fisher Scientific	MT21031CV
MES hydrate	Sigma Aldrich	M8250
Sodium Hydroxide Solution	Sigma Aldrich	S8263
Anhydrous sodium phosphate monobasic	Sigma Aldrich	S3139
Sodium Azide	Sigma Aldrich	S2002
**Biological Samples**		
Guinea Pig Complement	MP Biomedicals	08642831
**Experimental Models: Cell Lines**		
Human Peripheral Blood Leukopak (Tenth), Fresh	Stemcell Technologies	200-0092
Human Peripheral Blood Leukopak (Quarter), Fresh	Stemcell Technologies	70500.2

**Critical Commercial Assays**		
EZ-Link Sulfo NHS-LC-LC Biotin	Thermo Fisher	A35358
Fix & Perm Cell Permeabilization Kit (Medium A)	Thermo Fisher	GAS001S100
Fix & Perm Cell Permeabilization Kit (Medium B)	Thermo Fisher	GAS002S100
EasySep™ RBC Depletion Reagent	Stemcell Technologies	18170
EasySep™ Human NK Cell Isolation Kit	Stemcell Technologies	17955
EasySep™ Buffer	Stemcell Technologies	20144
Zebra-Spin Desalting and Chromatography Columns	Thermo Fisher	89882
**Software and Algorithms**		
iQue Forecyt 9.1	Sartorius	60028
R Studio V 4.5.1	R Project for Statistical Computing	RRID:SCR_000432
**Other**		
iQue Screener Plus	Sartorius	11811
Luminex™ xMAP INTELLIFLEX System	Thermo Fisher	APX2020
MagPlex Microspheres	DiaSorin	MC12001-01 (Cataloged by region)
FluoSpheres™ NeutrAvidin™-Labeled Microspheres, 1.0 µm, yellow-green fluorescent (505/515), 1% solids	Thermo Fisher	f8776
384-well HydroSpeed Plate Washer	Tecan	30190112
Countess™ 3 Automated Cell Counter	Thermo Fisher	AMQAX2000
Intelliflex Calibration Kit	Thermo Fisher	IFXCALK20
Intelliflex Performance Verification Kit	Thermo Fisher	IFXPVERK20
xMAP™ Sheath Concentrate PLUS, RUO	Thermo Fisher	4050023
iQue® Screener Plus Validation Beads	Sartorius	91091
iQue® Qsol Buffer Concentrate Solution	Sartorius	91304

Supplementary Figure 1

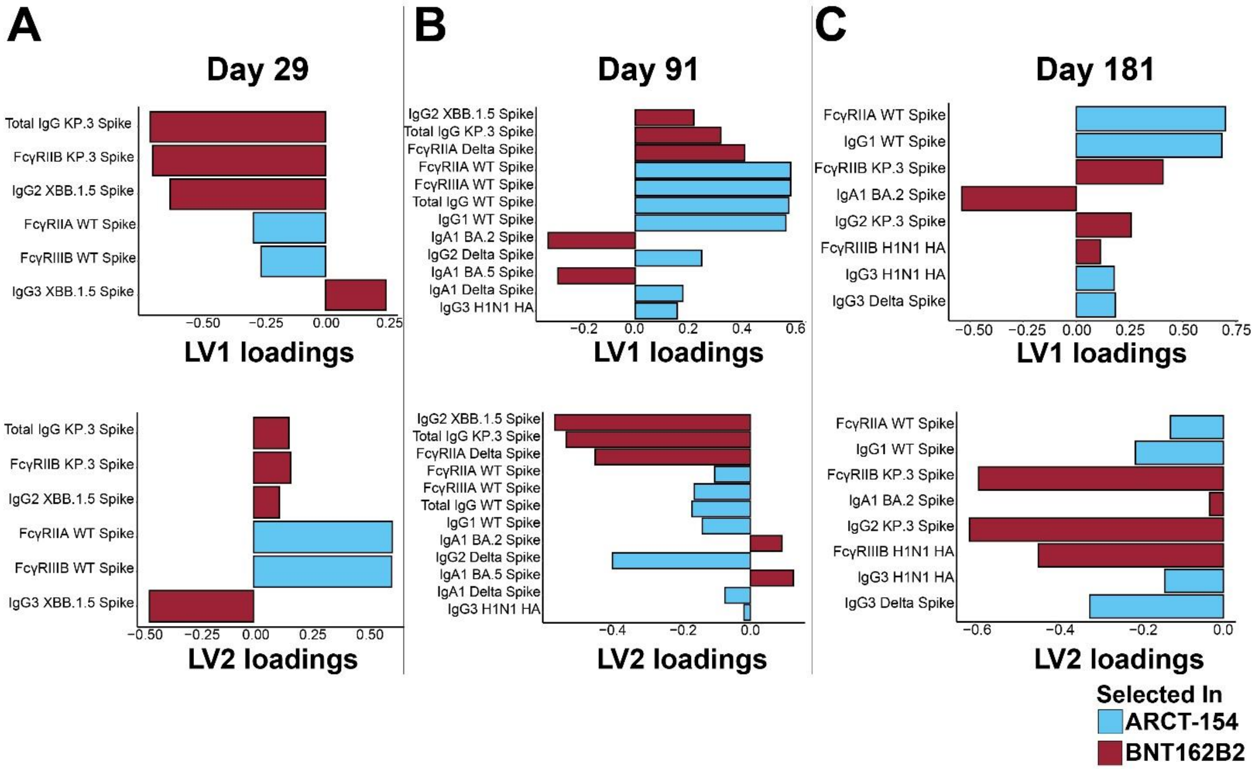

Supplementary Figure 1. Features driving separation through a least absolute shrinkage and selection operator (LASSO)- based PLSDA.

(A) LASSO-selected features for the PLSDA model at day 29 post-booster with BNT162B2 (red) and ARCT-154 (blue). Shown are the selected features and their scores on latent variable (LV) 1 (top) and LV2 (bottom). Shown in the bottom right corner is the color legend. No models that were statistically significant over random features and permuted labels could be made at day 1 and day 361, so no LASSO-features are shown for those time points.

(B) Same as A, but for the LASSO-selected features driving the model at day 91 post-booster.

(C) Same as A, but for the LASSO-selected features driving the model at day 181 post-booster.

Supplementary Figure 2

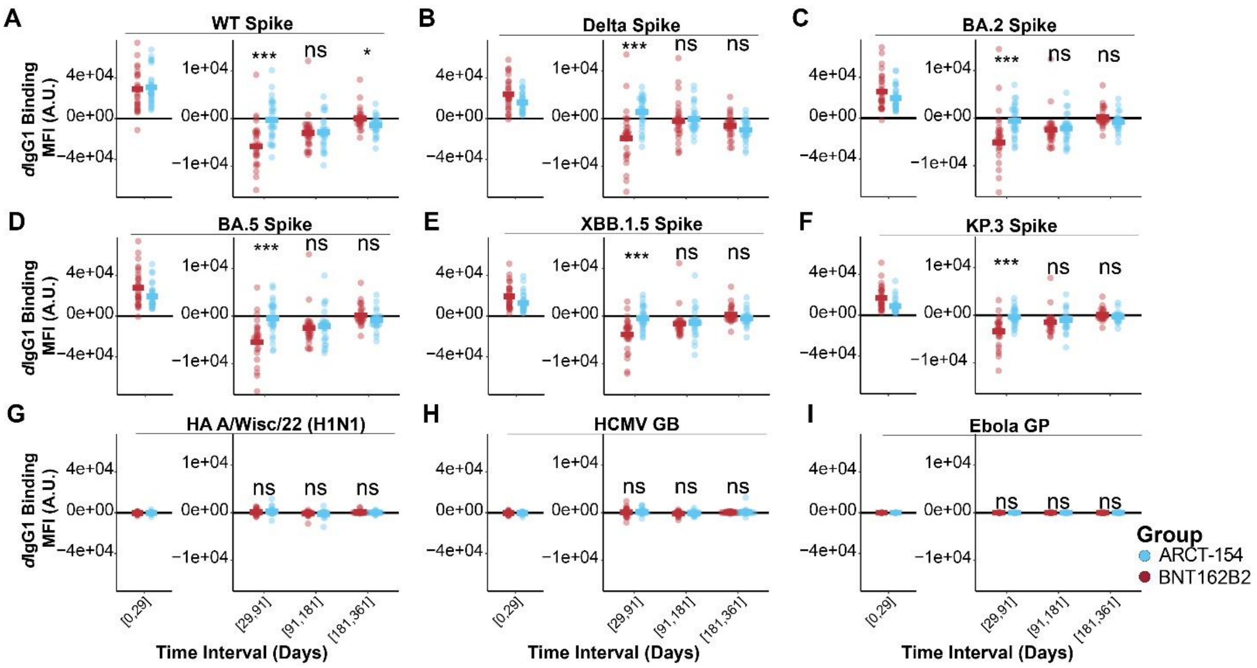

Supplementary Figure 2. IgG1 levels to target and target-related antigens have different rates of decay based on boosting with an mRNA- or an sa-mRNA-vaccine.

(A) Derivatives (rate of change, or *d*) were quantified for the time intervals shown on the x-axis for the two treatment arms (BNT162B2 in red, ARCT-154 in blue). Each dot represents the *d* of IgG1 to WT Spike at the time interval, and the colored horizontal bar indicates the group mean of *d* IgG1 WT Spike. A $d < 0$ indicates a negative slope, or contraction; a $d > 0$ indicates a positive slope, or expansion; and a *d* = indicates no change in slope, or sustained response. Statistical comparisons between the two groups were done for each time interval using a Wilcoxon Test followed by a false discovery rate (FDR) adjustment. Above each time interval, n.s. indicates not statistically significant after FDR correction ($p \geq 0.05$), * indicates $p < 0.05$ after FDR correction, ** indicates $p < 0.01$ after FDR correction, and *** indicates $p < 0.001$ after FDR correction. Acute phase stimulation comparisons were not performed as this was not a primary endpoint analysis (see Methods).

(B-F) Same as A, but for variant SARS-CoV-2 Spikes shown at the top of each graph.

(G-I) Same as A, but for high-exposure control off-target antigens (HA A/Wisc/22 H1N1 and HCMV GB), and for no-exposure control antigens (Ebola GP).

Supplementary Figure 3

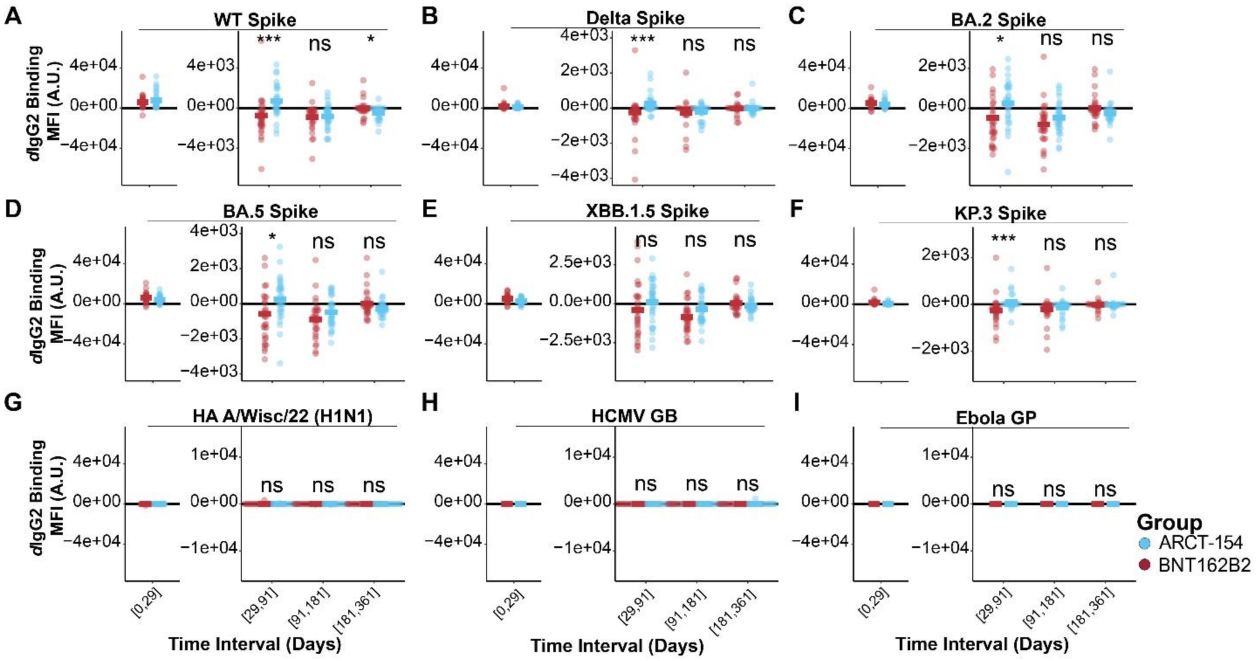

Supplementary Figure 3. IgG2 levels to target and target-related antigens have different rates of decay based on boosting with an mRNA- or an sa-mRNA-vaccine.

(A) Derivatives (rate of change, or *d*) were quantified for the time intervals shown on the x-axis for the two treatment arms (BNT162B2 in red, ARCT-154 in blue). Each dot represents the *d* of IgG2 to WT Spike at the time interval, and the colored horizontal bar indicates the group mean of *d* IgG2 WT Spike. A $d < 0$ indicates a negative slope, or contraction; a $d > 0$ indicates a positive slope, or expansion; and a *d* = indicates no change in slope, or sustained response. Statistical comparisons between the two groups were done for each time interval using a Wilcoxon Test followed by a false discovery rate (FDR) adjustment. Above each time interval, n.s. indicates not statistically significant after FDR correction ($p \geq 0.05$), * indicates $p < 0.05$ after FDR correction, ** indicates $p < 0.01$ after FDR correction, and *** indicates $p < 0.001$ after FDR correction. Acute phase stimulation comparisons were not performed as this was not a primary endpoint analysis (see Methods).

(B-F) Same as A, but for variant SARS-CoV-2 Spikes shown at the top of each graph.

(G-I) Same as A, but for high-exposure control off-target antigens (HA A/Wisc/22 H1N1 and HCMV GB), and for no-exposure control antigens (Ebola GP).

Supplementary Figure 4

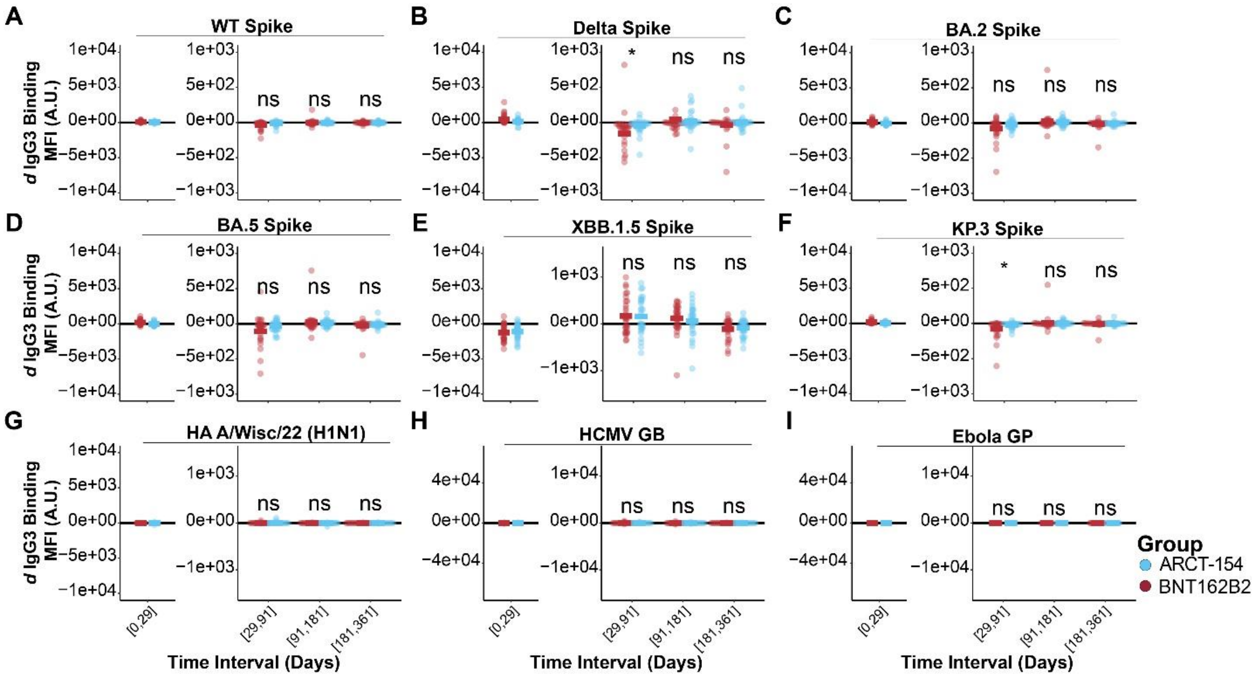

Supplementary Figure 4. IgG3 levels to target and target-related antigens have different rates of decay based on boosting with an mRNA- or an sa-mRNA-vaccine.

(A) Derivatives (rate of change, or *d*) were quantified for the time intervals shown on the x-axis for the two treatment arms (BNT162B2 in red, ARCT-154 in blue). Each dot represents the *d* of IgG3 to WT Spike at the time interval, and the colored horizontal bar indicates the group mean of *d* IgG3 WT Spike. A $d < 0$ indicates a negative slope, or contraction; a $d > 0$ indicates a positive slope, or expansion; and a *d* = indicates no change in slope, or sustained response. Statistical comparisons between the two groups were done for each time interval using a Wilcoxon Test followed by a false discovery rate (FDR) adjustment. Above each time interval, n.s. indicates not statistically significant after FDR correction ($p \geq 0.05$), * indicates $p < 0.05$ after FDR correction, ** indicates $p < 0.01$ after FDR correction, and *** indicates $p < 0.001$ after FDR correction. Acute phase stimulation comparisons were not performed as this was not a primary endpoint analysis (see Methods).

(B-F) Same as A, but for variant SARS-CoV-2 Spikes shown at the top of each graph.

(G-I) Same as A, but for high-exposure control off-target antigens (HA A/Wisc/22 H1N1 and HCMV GB), and for no-exposure control antigens (Ebola GP).

Supplementary Figure 5

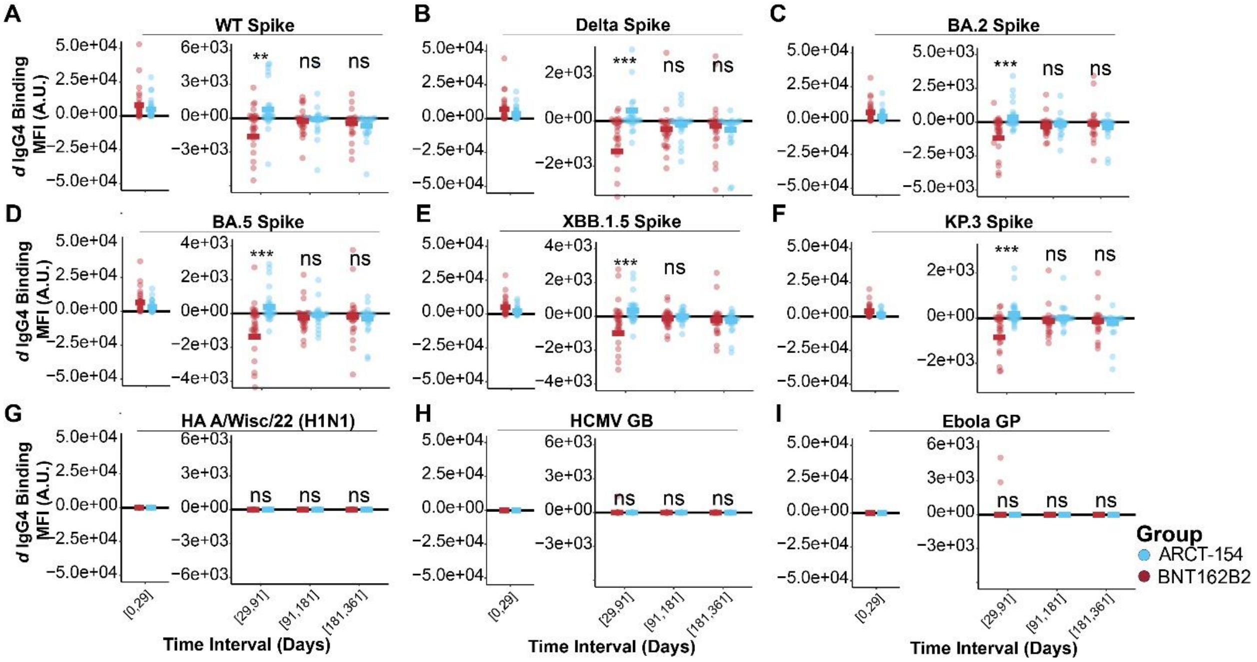

Supplementary Figure 5. IgG4 levels to target and target-related antigens have different rates of decay based on boosting with an mRNA- or an sa-mRNA-vaccine.

(A) Derivatives (rate of change, or *d*) were quantified for the time intervals shown on the x-axis for the two treatment arms (BNT162B2 in red, ARCT-154 in blue). Each dot represents the *d* of IgG4 to WT Spike at the time interval, and the colored horizontal bar indicates the group mean of *d* IgG4 WT Spike. A $d < 0$ indicates a negative slope, or contraction; a $d > 0$ indicates a positive slope, or expansion; and a $d =$ indicates no change in slope, or sustained response. Statistical comparisons between the two groups were done for each time interval using a Wilcoxon Test followed by a false discovery rate (FDR) adjustment. Above each time interval, n.s. indicates not statistically significant after FDR correction ($p \geq 0.05$), * indicates $p < 0.05$ after FDR correction, ** indicates $p < 0.01$ after FDR correction, and *** indicates $p < 0.001$ after FDR correction. Acute phase stimulation comparisons were not performed as this was not a primary endpoint analysis (see Methods).

(B-F) Same as A, but for variant SARS-CoV-2 Spikes shown at the top of each graph.

(G-I) Same as A, but for high-exposure control off-target antigens (HA A/Wisc/22 H1N1 and HCMV GB), and for no-exposure control antigens (Ebola GP).

Supplementary Figure 6

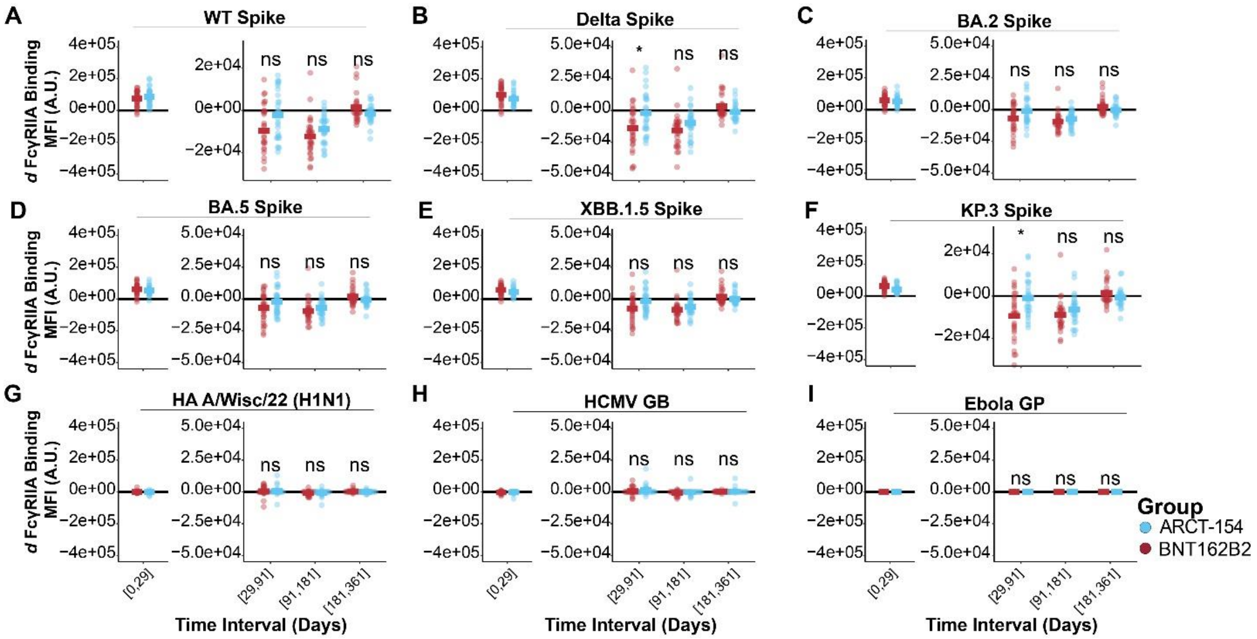

Supplementary Figure 6. FcγRIIA-binding antibody levels to target and target-related antigens have different rates of decay based on boosting with an mRNA- or an sa-mRNA-vaccine.

(A) Derivatives (rate of change, or *d*) were quantified for the time intervals shown on the x-axis for the two treatment arms (BNT162B2 in red, ARCT-154 in blue). Each dot represents the *d* of FcγRIIA-binding antibodies to WT Spike at the time interval, and the colored horizontal bar indicates the group mean of *d* FcγRIIA-binding antibodies to WT Spike. A $d < 0$ indicates a negative slope, or contraction; a $d > 0$ indicates a positive slope, or expansion; and a *d* = indicates no change in slope, or sustained response. Statistical comparisons between the two groups were done for each time interval using a Wilcoxon Test followed by a false discovery rate (FDR) adjustment. Above each time interval, n.s. indicates not statistically significant after FDR correction ($p \geq 0.05$), * indicates $p < 0.05$ after FDR correction, ** indicates $p < 0.01$ after FDR correction, and *** indicates $p < 0.001$ after FDR correction. Acute phase stimulation comparisons were not performed as this was not a primary endpoint analysis (see Methods).

(B-F) Same as A, but for variant SARS-CoV-2 Spikes shown at the top of each graph.

(G-I) Same as A, but for high-exposure control off-target antigens (HA A/Wisc/22 H1N1 and HCMV GB), and for no-exposure control antigens (Ebola GP).

Supplementary Figure 7

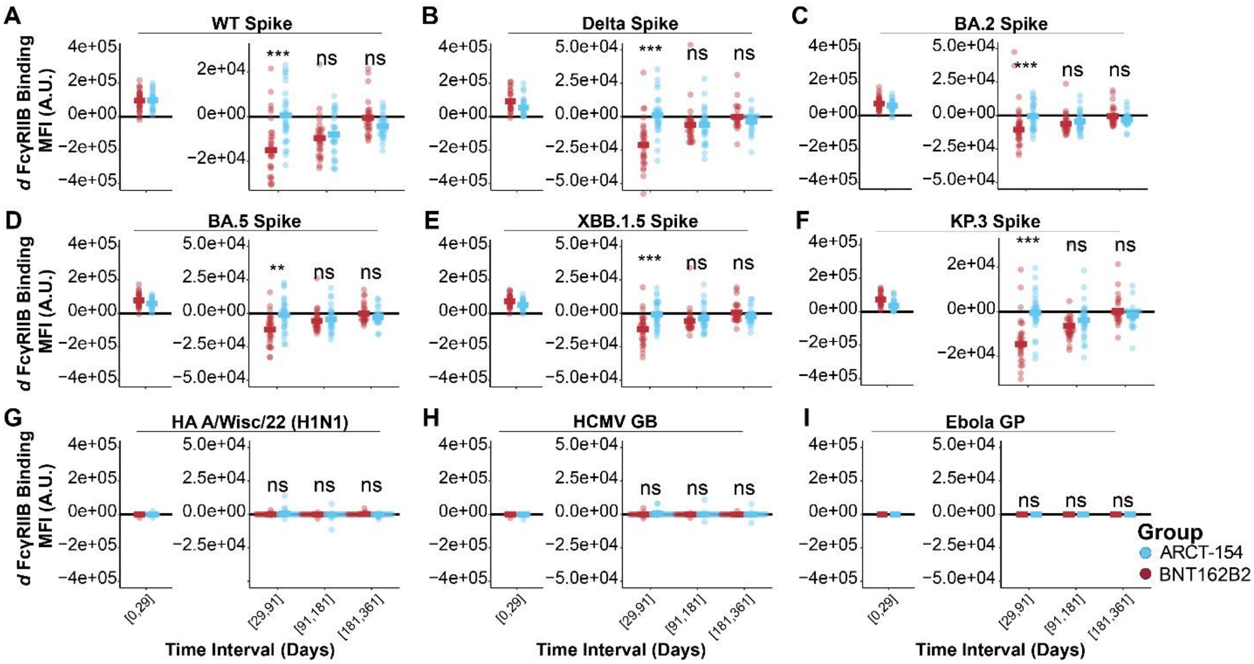

Supplementary Figure 7. FcγRIIB-binding antibody levels to target and target-related antigens have different rates of decay based on boosting with an mRNA- or an sa-mRNA-vaccine.

(A) Derivatives (rate of change, or *d*) were quantified for the time intervals shown on the x-axis for the two treatment arms (BNT162B2 in red, ARCT-154 in blue). Each dot represents the *d* of FcγRIIB-binding antibodies to WT Spike at the time interval, and the colored horizontal bar indicates the group mean of *d* FcγRIIB-binding antibodies to WT Spike. A $d < 0$ indicates a negative slope, or contraction; a $d > 0$ indicates a positive slope, or expansion; and a *d* = indicates no change in slope, or sustained response. Statistical comparisons between the two groups were done for each time interval using a Wilcoxon Test followed by a false discovery rate (FDR) adjustment. Above each time interval, n.s. indicates not statistically significant after FDR correction ($p \geq 0.05$), * indicates $p < 0.05$ after FDR correction, ** indicates $p < 0.01$ after FDR correction, and *** indicates $p < 0.001$ after FDR correction. Acute phase stimulation comparisons were not performed as this was not a primary endpoint analysis (see Methods).

(B-F) Same as A, but for variant SARS-CoV-2 Spikes shown at the top of each graph.

(G-I) Same as A, but for high-exposure control off-target antigens (HA A/Wisc/22 H1N1 and HCMV GB), and for no-exposure control antigens (Ebola GP).

Supplementary Figure 8

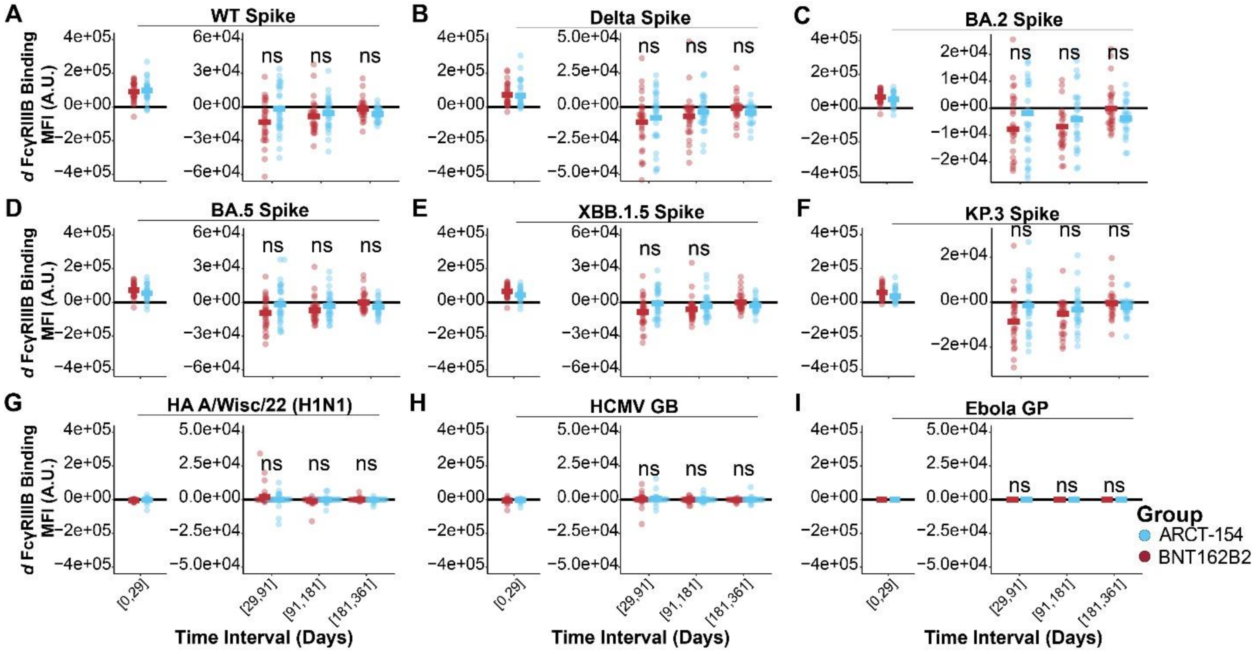

Supplementary Figure 8. FcγRIIIB-binding antibody levels to target and target-related antigens have different rates of decay based on boosting with an mRNA- or an sa-mRNA-vaccine.

(A) Derivatives (rate of change, or *d*) were quantified for the time intervals shown on the x-axis for the two treatment arms (BNT162B2 in red, ARCT-154 in blue). Each dot represents the *d* of FcγRIIIB-binding antibodies to WT Spike at the time interval, and the colored horizontal bar indicates the group mean of *d* FcγRIIIB-binding antibodies to WT Spike. A $d < 0$ indicates a negative slope, or contraction; a $d > 0$ indicates a positive slope, or expansion; and a $d =$ indicates no change in slope, or sustained response. Statistical comparisons between the two groups were done for each time interval using a Wilcoxon Test followed by a false discovery rate (FDR) adjustment. Above each time interval, n.s. indicates not statistically significant after FDR correction ($p \geq 0.05$), * indicates $p < 0.05$ after FDR correction, ** indicates $p < 0.01$ after FDR correction, and *** indicates $p < 0.001$ after FDR correction. Acute phase stimulation comparisons were not performed as this was not a primary endpoint analysis (see Methods).

(B-F) Same as A, but for variant SARS-CoV-2 Spikes shown at the top of each graph.

(G-I) Same as A, but for high-exposure control off-target antigens (HA A/Wisc/22 H1N1 and HCMV GB), and for no-exposure control antigens (Ebola GP).

Supplementary Figure 9

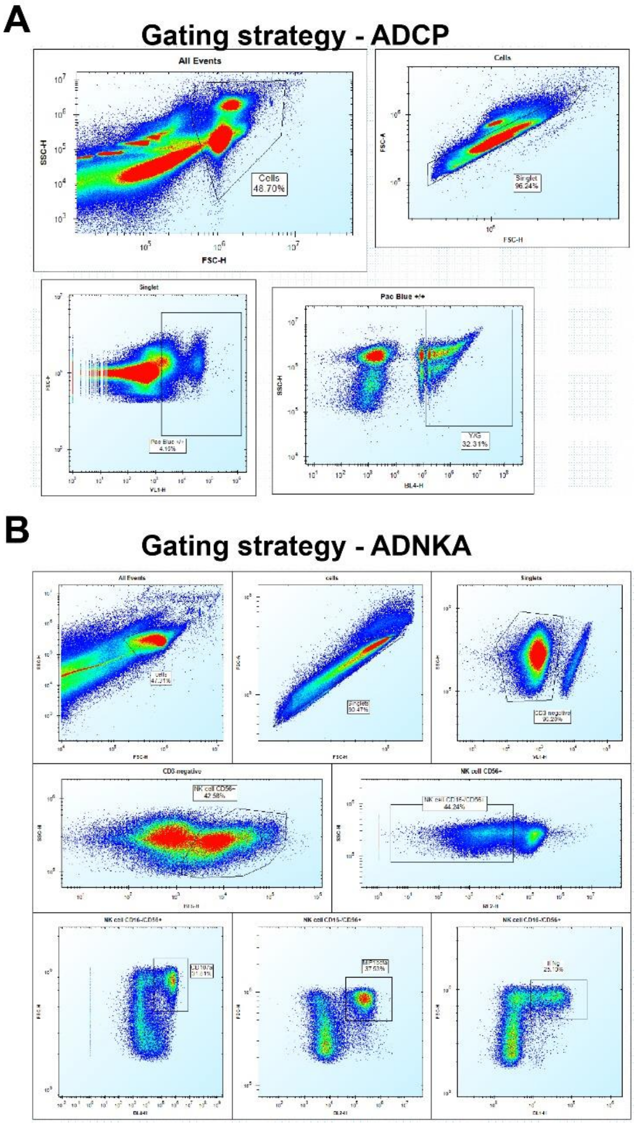

Supplementary Figure 9. Flow cytometry gating for antibody effector assays.

(A) Gating strategy for ADCP from primary-derived human leukopacks. Singlets were gated, and CD14+ cells were further gated to quantify monocyte responses.

(B) Gating strategy for ADNKA from primary-derived human leukopacks. Singlets were gated, and CD56+/CD3- NK cells were further gated to quantify NK responses.
